# New Appliances and Things Medical

**Published:** 1902-08-09

**Authors:** 


### NEW APPLIANCES AND THINGS MEDICAL.

### OOWANA: A NEW TOILET SOAP.

(The Oowana Soap Company, Limited, 205 Victoria Street, London, S.W.)

In certain respects this new toilet soap presents features which are unusual in the manufacture of even the best varieties of soap; it possesses great cleansing properties, and produces an excellent lather without the great excess of free alkali whereby these desirable results are usually secured. The manufacturers claim to effect their objects by the substitution of a vegetable extract for the excess of alkali. It is further stated that this soap contains no animal matter, a statement which means, we presume, that it contains no nitrogeneous animal matter, and not that the fats employed are of vegetable origin. We have made trial of this soap and find it fulfills the conditions claimed for it; it is deliciously scented, and is free from all ingredients likely to prove in the least deleterious to the most delicate skins.

### A NEW DISINFECTING FLUID.

(The Nobes Disinfecting Company, 4 Eastcheap, London, E.C.)

This new disinfectant is a powerful oxidising and deodorising fluid; it supplies in a practical form and in a condition ready for immediate and safe use, an antiseptic chemical body which is very highly appreciated in the scientific world. We commend Nobes disinfectant to the notice of those who appreciate the application to domestic hygiene of scientific antiseptic principles of the highest order.

